# BCG Disease in SCID: Three Decades of Experience in a Pediatric Transplant Center

**DOI:** 10.1007/s10875-021-01143-y

**Published:** 2021-10-07

**Authors:** Nicoletta Cocchi, Eva-Maria Jacobsen, Manfred Hoenig, Ansgar Schulz, Catharina Schuetz

**Affiliations:** 1 Department of Pediatrics, Medical Center Dritter Orden, Munich, Germany; 2 Department of Pediatrics, University Medical Center Ulm, Ulm, Germany; 3 Department of Pediatrics, Medizinische Fakultät Carl Gustav Carus, Technische Universität, Dresden, Germany; 4 Klinik und Poliklinik für Kinder- und Jugendmedizin, Universitätsklinikum Carl Gustav Carus an der Technischen Universität Dresden, Fetscherstrasse 74, 01307 Dresden, Germany

To the editor

Patients with severe combined immunodeficiency (SCID) have an increased incidence of BCG disease following vaccination compared to other infants [1]. BCG disease is classified in local, regional, distant, and disseminated [2]. Disease flares often occur following hematopoietic stem cell transplantation (HSCT). Consequently, management of BCG disease in SCID patients undergoing HSCT remains a challenge, and optimal management is not standardized.

We retrospectively analyzed the clinical course of BCG disease in 36 SCID patients who underwent HSCT at the Department of Pediatrics at the University Medical Center Ulm between 1983 and 2014, focusing on the impact of immune reconstitution and tuberculostatic therapy. All patients had a documented BCG vaccination. The study was approved by the ethical committee of Ulm University. Furthermore, we collected information via a standardized questionnaire about current BCG disease management in 15 international centers associated with the European Society for Blood and Marrow Transplantation (EBMT). Results from the international survey can be found in Table 1 of the supplemental online material.

Main clinical and epidemiological characteristics of the cohort are summarized in Table 1. Although BCG strains were not documented in our patient files, we were able to map the strains using http://BCGatlas.org, which allows identification of most strains according to year of vaccination and location. Not for all, but for 28/36 patients we managed to infer the BCG strain (Table 2, supplemental online material): respectively Copenhagen 1331 (25 children from Germany, Spain, and Latvia), Moreau (1 child from Croatia), Tokyo-172 (1 child from Portugal), Pasteur (1 child from Bosnia). For UAE and Morocco there are no official data available, whereas in Saudi Arabia three strains were in use (Copenhagen 1331, Pasteur, Tokyo-172). The Copenhagen 1331 strain is classified by the WHO as a “strong” one (WHO, Recommendations to assure the quality, safety and efficacy of BCG vaccines, Annex 3, 2013). We then compared time to remission from BCG disease in patients who received Copenhagen 1331 (*n* = 25) with all others (*n* = 11). Surprisingly, time to remission was significantly longer in patients following vaccination with the Copenhagen 1331 strain as shown in Table 2 of the supplements (464 days post HSCT vs 191 days, *p* = 0.01).

**Table 1:** Main epidemiological and clinical characteristics of 36 SCID patients

	*N*^o^	Gender	Age at SCID diagnosis (mo)	Age at first BCG disease manifestation (mo)	Age at HSCT (mo)	Donor	Duration antimycobacterial therapy (mo)	Time to remission from BCG disease (d)	Deaths
No BCG disease symptoms	3	F *n* = 1, M *n* = 2	3.7 (1–7)	-	8 (2–16)	Haplo-id., *n* = 3	7 (3–14)	-	0
Locoregional BCG disease	10	F *n* = 4, M *n* = 6	4.1* (2–5.5)	5.7 (2–10)	6.1† (3.5-11)	Haplo-id., *n* = 7; HLA-id., *n* = 3	25.3 (2–84)	220^‡^	2
Disseminated BCG disease	23	F *n* = 6, M *n* = 17	5.8* (2–12)	5.5 (2–14), *n* = 2 n.a.	8.8† (4–16)	Haplo-id., *n* = 17; HLA-id., *n* = 6	39.6 (4–86), *n* = 2 n.a.	458‡, *n* = 2 n.a., *n* = 5 n.r.	8

*F* female, *M* male, *HSCT* hematopoietic stem cell transplantation, *n.a.* data not available, *n.r.* not reached. *Comparison of age at SCID diagnosis between patients with locoregional and disseminated BCG disease: *p* = 0.01. †Comparison of age at HSCT between patients with locoregional and disseminated BCG disease: *p* = 0.02. ‡Comparison of time to remission in days after HSCT between patients with locoregional and disseminated BCG disease: *p* = 0.04

Molecular genetics of SCID variants were determined in 26 patients (72%): *IL2RG* deficiency and *RAG1/RAG2* defects (including Omenn syndrome) were the most frequently reported, followed by Artemis (*DCLRE1C*), *JAK3*, *ADA*, *AK2*, *IL7RA*, *ZAP70*, and *CD3ε*. We analyzed the distribution of BCG disease subtype on the basis of immunophenotype (T^−^B^+^ versus T^−^B^−^ SCID). There was no difference between these groups as for distribution of disease subtype. There was no statistical significance of BCG disease subtype with respect to the presence of T cells or MFT. Most infants presented with a combination of respiratory tract infections, enteritis, and failure to thrive. Rotavirus was detected in 12 patients, whereas 7 children were infected with cytomegalovirus (CMV). BCG disease was often one of the initial manifestations of immunodeficiency: 24 patients developed symptoms of BCG disease before or concomitantly with SCID diagnosis. In our cohort, most patients who presented with first BCG disease symptoms prior 4 months of age developed disseminated disease, though these results were not statistically significant (the 3 asymptomatic patients were excluded). The most common BCG disease manifestations were inflammation at the vaccination site, lymphadenopathy, skin rash, and fever. Central nervous system involvement and arthritis were exceptional. Ten of the 36 patients died after HSCT due to various complications (age range: 7 months to 12 years). One death was directly related to pulmonary and central nervous system BCG disease; in another patient, BCG pneumonia with respiratory insufficiency was suspected. In those patients with disseminated BCG disease (*n* = 23/36), SCID was diagnosed with significant delay, and HSCT was performed later as compared to patients with locoregional disease (median age at SCID diagnosis respectively 5.8 and 4.1 months, *p* = 0.01; median age at HSCT respectively 8.8 and 6.1 months, *p* = 0.02).

All patients were treated with tuberculostatic drugs. The majority (*n* = 30/36) received a combination therapy (3–5 drugs). Patients with locoregional BCG disease were most often treated (*n* = 7/10) with an antimycobacterial triple therapy, whereas the majority of patients with disseminated BCG disease were treated with four or more tuberculostatic drugs (19/23 with four or more drugs, 4/23 with three drugs). Similar therapeutic strategies were reported from 15 EBMT centers: for locoregional disease, 2 (53%) or 3 (40%) tuberculostatic agents would be administered, whereas 3 (46%) or more drugs (47%) for disseminated disease. The most common combination treatment used in our cohort was rifampicin, isoniazid plus ethambutol. Add-on drugs were mostly quinolones and aminoglycosides. Antimycobacterial therapy was started concomitantly (*n* = 14/36) or after (*n* = 18/36) SCID diagnosis and in most cases (*n* = 29/36) before HSCT was performed. Administration of tuberculostatic treatment more than 6 weeks prior to HSCT correlated with absence of BCG disease symptoms 1 year following HSCT (*p* = 0.006). Among patients with complete remission of BCG disease at 12 months’ follow-up (*n* = 18), 5 had no BCG disease symptoms at first medical assessment. Patients with symptoms at 12 months’ follow-up (*n* = 10) had all been symptomatic for BCG disease from the beginning; hence, absence of BCG disease symptoms at first medical assessment correlates with a better outcome. However, these results were not statistically significant.

Tuberculostatic drugs were administrated on average for 12 months after HSCT (range: 1–31 months), depending on the clinical course and immune reconstitution. This was in line with the results from our international survey, as tuberculostatic therapy is applied for 12 or more months in most centers (9/15).

HSCT was performed in all patients to cure underlying SCID (Table 3a/3b, supplemental online material). Haploidentical or mismatched familiar donors were available for the majority of children (*n* = 26), whereas matched familiar and matched unrelated donor could be identified for 7 and 3 patients respectively. A total of 34/36 patients received GvHD prophylaxis via T cell depletion of the graft, and/or immunosuppressive drugs. The large majority (25/26) of haploidentical grafts had been CD34 + enriched and T cell depleted via lectin-agglutination and erythrocyte-rosette depletion or by immunomagnetic methods. One patient showed HLA homozygosity for three loci (A, B, and C) and was HLA-identical in the DRB1 locus with his mother; hence, this graft was not manipulated before transplant. Four patients received myeloablative conditioning before transplantation: 18 with reduced intensity conditioning (RIC) and/or immunotherapy (IT), and 14 without conditioning. There were no differences in time to remission from BCG disease in patients who received any kind of conditioning versus no conditioning at all. After HSCT, 21 patients developed acute GvHD (15 following haploidentical and 6 following HLA-identical transplantation), which resulted in chronic GvHD in 6 of them. Patients who underwent haploidentical HSCT or were treated with immunosuppressive therapy due to GvHD presented more often with locoregional as compared to disseminated BCG disease, but this finding was not statistically significant. Time to remission from BCG disease did not substantially differ between haploidentical or HLA-identical transplantation. In our international survey, nine centers would administer not only tuberculostatic but also immunosuppressive treatment to better control BCG disease flares. Presence of BCG disease, however, would not influence donor choice, and HLA-identical donors are mostly preferred.

BCG disease worsened in 27/36 patients following HSCT (Fig. 1, supplemental material). Exacerbation occurred either early after HSCT (2–16 days) in 9 patients or concomitantly with T cell immune reconstitution (defined as CD3 + T cells > 500/μL) in 18 patients. In 3 of these late occurrences, the Mantoux test turned positive during disease flare. Early exacerbation, which is rarely described in the literature, occurred mainly after transplantation from HLA-identical donors.

Cure from BCG disease was documented in 27/36 patients or 75% (median time of absence of BCG disease signs and symptoms: 380 days after HSCT, range 14–930). Locoregional disease resolved significantly earlier than disseminated BCGitis (220 and 458 days respectively, *p* = 0.03).

In the course of the 30 years analyzed, distribution of BCG disease subtype or time to remission from BCG disease remained consistent. It is however worth mentioning that all deaths occurred before 1995.

Our data emphasize the importance of early SCID diagnosis and HSCT to prevent BCG disease outbreak and/or severe BCG disease in patients with a history of BCG vaccination, as previously observed [1, 3, 4]. Although no risk factors for developing severe (i.e., disseminated) BCG disease were identified when analyzing data according to underlying SCID gene variant or immunophenotype, time of treatment plays an important role in management of BCG disease. Patients presenting BCG disease symptoms early in life (< 4 months) seem to have a higher risk for dissemination. This finding needs to be verified in an even larger cohort. Initiating tuberculostatic treatment at the time of SCID diagnosis—even in the absence of BCG symptoms—may protect from BCG complications [1]. Furthermore, our results suggest that tuberculostatic treatment initiated prior HSCT is usually associated with better prognosis 1 year after HSCT. Data about whether preemptive treatment with a single antimycobacterial agent versus combination therapy is indicated is inconclusive. However, there are reports that routine administration of isoniazid alone was not effective in preventing BCG dissemination in BCG-vaccinated SCID patients (Verzegnassi Fiorotte et al., Poster Session ESID Meeting 2020, unpublished data). Careful observation is always recommended in the absence of BCG symptoms [5].

BCG-vaccinated SCID patients who undergo HSCT are at high risk for exacerbation of BCG disease. This may occur concomitantly with immune reconstitution shown by tuberculin test positivity [1], reminiscent of IRIS (immune reconstitution inflammatory syndrome) in HIV-infected individuals started on antiretroviral therapy [2]. In presence of T-replete grafts, exacerbation can occur few days after transplantation. Whether T cell reduction may prevent or dampen inflammatory response in the context of immune reconstitution remains an open question. The use of immunosuppressive drugs to control disease flare can be discussed and is currently adopted in some centers (results from international survey, Table 1 supplemental online material).

In conclusion, HSCT is curative for most SCID patients with BCG disease. Early initiation of tuberculostatic treatment prior HSCT is beneficial. We recommend early combination of antimycobacterial drugs (≥ 2) prior HSCT in BCG-vaccinated SCID babies including for locoregional disease or asymptomatic infants. Moreover, timely HSCT should be encouraged as it is associated with less severe, i.e., locoregional, disease (*p* = 0.03).

Identifying BCG disease flares, which frequently occur following HSCT, is necessary in order to intensify tuberculostatic therapy; furthermore, adjuvant immunosuppressive therapy may also be useful.

Delaying BCG vaccination is key for preventing BCG disease in undiagnosed SCID babies. With the expanding availability of TREC screening for T cell lymphopenia in newborns, timing of BCG vaccination should be reevaluated by local health authorities, and no longer be administered right after birth.

## Supplementary Information

Below is the link to the electronic supplementary material.

Supplementary file1 (PDF 789 KB)

Supplementary file2 (DOCX 80.4 KB)

Supplementary file3 (DOCX 22.6 KB)

Supplementary file4 (PDF 893 KB)
