# Supplementary material for: BCG Disease in SCID: Three Decades of Experience in a Pediatric Transplant Center
Source: J Clin Immunol. 2021 Oct 7;42(1):195–8. doi: 10.1007/s10875-021-01143-y (PMC8821078; doi:10.1007/s10875-021-01143-y)
Supplement: Supplementary file 1 — Supplementary file1 (PDF 789 KB) [file 10875_2021_1143_MOESM1_ESM.pdf]

BCG disease in SCID: three decades of experience in a pediatric transplant center

Nicoletta Cocchi[1,2], Eva-Maria Jacobsen[2], Manfred Hoenig[2], Ansgar Schulz[2], Catharina Schuetz[2,3]

1. Medical Center Dritter Orden, Department of Pediatrics, Munich, Germany;
2. University Medical Center Ulm, Department of Pediatrics, Ulm, Germany;
3. Department of Pediatrics, Medizinische Fakultät Carl Gustav Carus, Technische Universität Dresden, Germany

Corresponding author: Catharina Schütz, MD
Klinik und Poliklinik für Kinder- und Jugendmedizin
Universitätsklinikum Carl Gustav Carus an der TU Dresden
Fetscherstrasse 74
D-01307 Dresden
Germany
catharina.schuetz@ukdd.de
+49 351 458 11702
+49 351 458 4384

Supplemental material: Figure 1

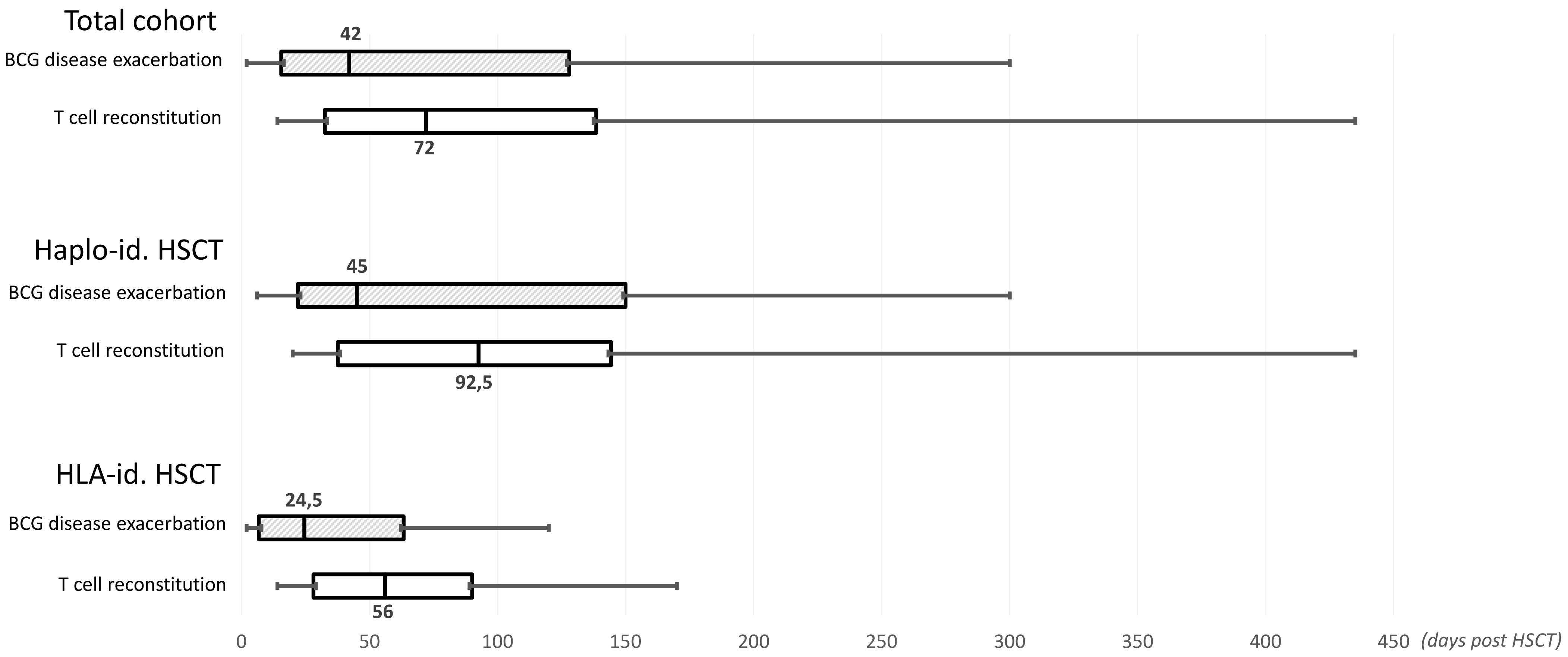

Figure 1. Timepoint of BCG disease exacerbation and T cell-immune reconstitution in days after hematopoietic stem cell transplantation with median values. HSCT: hematopoietic stem cell transplantation; Haplo-id. HSCT: patients who underwent haploidentical HSCT; HLA-id. HSCT: patients who underwent HLA-identical HSCT; T cell- immune reconstitution was defined as CD3+ T cells >500/µl.
