# Supplementary material for: BCG Disease in SCID: Three Decades of Experience in a Pediatric Transplant Center
Source: J Clin Immunol. 2021 Oct 7;42(1):195–8. doi: 10.1007/s10875-021-01143-y (PMC8821078; doi:10.1007/s10875-021-01143-y)
Supplement: Supplementary file 2 — Supplementary file2 (DOCX 80.4 KB) [file 10875_2021_1143_MOESM2_ESM.docx]

**BCG disease in SCID: three decades of experience in a pediatric transplant center**

Nicoletta Cocchi^1,2^, Eva-Maria Jacobsen^2^, Manfred Hoenig^2^, Ansgar Schulz^2^, Catharina Schuetz^2,3^

1. Medical Center Dritter Orden, Department of Pediatrics, Munich, Germany;
2. University Medical Center Ulm, Department of Pediatrics, Ulm, Germany;
3. Department of Pediatrics, Medizinische Fakultät Carl Gustav Carus, Technische Universität Dresden, Germany

Corresponding author: Catharina Schuetz, MD

Klinik und Poliklinik für Kinder- und Jugendmedizin

Universitätsklinikum Carl Gustav Carus an der TU Dresden

Fetscherstrasse 74

D-01307 Dresden

Germany

[catharina.schuetz@ukdd.de](mailto:catharina.schuetz@ukdd.de)

+49 351 458 11702

+49 351 458 4384

**Supplemental material: Table 1**

**Table 1. Results from international survey about BCG disease management**

**no n=12**

**no n=13**

**yes n=6**

**12 months n=7**

**at least 6 months n=5**

**> 12 months n=2**

**depends on clinical status and immune reconstitution n=1**

**add immunosuppressive and tuberculostatic drugs n=6**

**add tuberculostatic drugs n=5**

**add immunosuppressive drugs n=3**

**don’t know n=1**

**don’t know n=1**

**yes n=1**

**yes n=1**

**don’t know n=1**

**≥ 3 drugs n=7**

**know n=1**

**triple therapy n=7**

**don’t know n=1**

**double therapy n=8**

**triple therapy n=6**

**don’t know n=1**

**no n=9**
