# Supplementary material for: BCG Disease in SCID: Three Decades of Experience in a Pediatric Transplant Center
Source: J Clin Immunol. 2021 Oct 7;42(1):195–8. doi: 10.1007/s10875-021-01143-y (PMC8821078; doi:10.1007/s10875-021-01143-y)
Supplement: Supplementary file 3 — Supplementary file3 (DOCX 22.6 KB) [file 10875_2021_1143_MOESM3_ESM.docx]

**BCG disease in SCID: three decades of experience in a pediatric transplant center**

Nicoletta Cocchi^1,2^, Eva-Maria Jacobsen^2^, Manfred Hoenig^2^, Ansgar Schulz^2^, Catharina Schuetz^2,3^

1. Medical Center Dritter Orden, Department of Pediatrics, Munich, Germany;
2. University Medical Center Ulm, Department of Pediatrics, Ulm, Germany;
3. Department of Pediatrics, Medizinische Fakultät Carl Gustav Carus, Technische Universität Dresden, Germany

Corresponding author: Catharina Schuetz, MD

Klinik und Poliklinik für Kinder- und Jugendmedizin

Universitätsklinikum Carl Gustav Carus an der TU Dresden

Fetscherstrasse 74

D-01307 Dresden

Germany

[catharina.schuetz@ukdd.de](mailto:catharina.schuetz@ukdd.de)

+49 351 458 11702

+49 351 458 4384

**Supplemental material: Table 2**

**Table 2. BCG strains used for vaccination in our cohort**

|  | Copenhagen 1331 | Tokyo-172 | Moreau | Pasteur | Unknown |
| --- | --- | --- | --- | --- | --- |
| N^o^. of patients | n=25 | n=1 | n=1 | n=1 | n=8 |

| No symptoms | n=3 | n=0 |
| --- | --- | --- |
| Locoregional disease | n=7 | n=3 |
| Disseminated disease | n=17 | n=8 |
| Time to remission  (days after HSCT) | 464d***** | 191d***** |

*Comparison of time to remission from BCG disease in days after HSCT: ***p*=0.01**. Reference for BCG strains: <http://www.bcgatlas.org/>, visited on 30^th^ July 2021
