# Supplementary material for: BCG Disease in SCID: Three Decades of Experience in a Pediatric Transplant Center
Source: J Clin Immunol. 2021 Oct 7;42(1):195–8. doi: 10.1007/s10875-021-01143-y (PMC8821078; doi:10.1007/s10875-021-01143-y)
Supplement: Supplementary file 4 — Supplementary file4 (PDF 893 KB) [file 10875_2021_1143_MOESM4_ESM.pdf]

BCG disease in SCID: three decades of experience in a pediatric transplant center

Nicoletta Cocchi[1,2], Eva-Maria Jacobsen[2], Manfred Hoenig[2], Ansgar Schulz[2], Catharina Schuetz[2,3]

1. Medical Center Dritter Orden, Department of Pediatrics, Munich, Germany;
2. University Medical Center Ulm, Department of Pediatrics, Ulm, Germany;
3. Department of Pediatrics, Medizinische Fakultät Carl Gustav Carus, Technische Universität Dresden, Germany

Corresponding author: Catharina Schuetz, MD
Klinik und Poliklinik für Kinder- und Jugendmedizin
Universitätsklinikum Carl Gustav Carus an der TU Dresden
Fetscherstrasse 74
D-01307 Dresden
Germany
catharina.schuetz@ukdd.de
+49 351 458 11702
+49 351 458 4384

Supplemental material: Table 3a/3b

Table 3a. HSCT procedures in haploidentical transplantations and GvHD

Pat	Age at HSCT	Genetics	Donor	Graft	T cell depletion	Conditioning	Serotherapy	GvHD prophylaxis (IS drugs)	Acute GvHD	Chronic GvHD
1	11	RAG	Father	PBSC	CD34+ CD2-	**MA** (BU16/FLU160)	ATG,ALEM	-	-	-
4	11	JAK3	Mother	PBSC	CD34+ CD2-	**MA** (BU16/FLU160)	-	-	-	-
5	7	XL, γc	Mother	PBSC	CD34+	-	ATG	CSA	-	-
6	16	IL2RG	Mother	PBSC	CD34+ CD2-	-	-	-	-	-
7	2	IL2RG	Mother	BM	E-rosetting	**RIC** (CY120/BU8)	-	-	I° (skin)	-
8	13	JAK3	Mother	PBSC	CD34+ CD2-	**MA** (BU12,8)	-	-	II° (skin, liver)	-
10	16	XL, γc	Father	PBSC/BM *	E-rosetting	*	*	CSA*	I° (skin)	I° (skin)
12	6,5	RAG1	Mother	BM	E-rosetting	**RIC** (CY/TT) *dosis n.a.*	-	-	-	-
13[%]	7	IL2RG	Father	BM	E-rosetting	-	-	*n.a.*	I° (skin)	-
14	6	IL7RA	Father	PBSC	E-rosetting	**RIC** (CY200/BU8)	-	-	-	-
18	7	CD3ε	Mother	PBSC	CD34+ CD2- CD3-	**RIC** (BU8/FLU200)	ATG	CSA	I° (skin)	-
19	4	RAG1	Mother	PBSC	CD34+ CD2-	**RIC** (BU12/FLU5)	ATG	CSA	IV° (skin)	-
20	5	RAG	Father	BM	E-rosetting	**RIC** (CY200/BU8)	-	-	II° (skin, liver)	II° (skin, liver)

Table 3a. HSCT procedures in haploidentical transplantations and GvHD

Patient	Age at HSCT	Genetics	Donor	Graft	T cell depletion	Conditioning	Serotherapy	GvHD prophylaxis (IS drugs)	Acute GvHD	Chronic GvHD
21	7	Artemis	Father	BM	E-rosetting	-	-	CSA before 3. HSCT	-	-
22	8	Unknown (B-, MFT)	Mother	BM	E-rosetting	**RIC** (BU/CY) *dosis n.a.*	ATG	-	II° (skin)	-
23	6,5	Unknown (B-)	Mother	BM	E-rosetting	-	-	CSA	I° (skin)	-
24	8	XL, γc	Mother	BM	E-rosetting	**RIC** (CY200/BU8)	-	-	I° (skin)	-
25	10	Unknown (B- NK+,MFT)	Mother	BM	E-rosetting	**RIC** (BU8 for 4d)	-	-	I° (skin)	II° (skin, liver, gut)
27	13	IL2RG	Mother	BM	E-rosetting	-	-	-	-	-
28	8	Artemis	Mother	BM	- †	-	-	PDN	III° (skin, liver)	-
29	8	Unknown (B+, MFT)	Mother	BM	E-rosetting	**RIC** (CY200/TT40)	-	-	I° (skin)	Severe (liver, gut)
31	6	RAG1	Mother	BM	E-rosetting	**RIC**(CY200/BU8)	ATG	PDN	-	-
32	5,5	ADA	Father	BM	E-rosetting	**RIC** (CY200/BU8)	ATG	-	II° (skin)	II° (skin, liver, gut)
34	10	unknown (B+)	Father	BM	E-rosetting	-	-	-	I° (skin)	-
35	8	Unknown (B+NK-)	Mother	BM	E-rosetting	-	-	-	-	-
36	11	Unknown (B-NK+)	Father	BM	E-rosetting	-	-	-	-	-

Table 3b. HSCT procedures in HLA-identical transplantations and GvHD

Patient	Age at HSCT (mo)	Genetics	Donor	Graft	T cell depletion	Conditioning	Serotherapy	GvHD prophylaxis (IS drugs)	Acute GvHD	Chronic GvHD
2	4	AK2	Father	BM	-	**RIC** (TREO36)	-	CSA	II° (skin)	-
3	4	RAG1	MUD	BM	-	**RIC** (FLU120/TREO36)	ALEM	CSA, MMF	I° (skin)	-
9	4	Unknown (B-)	Brother	BM	-	-	-	-	-	-
11	10	IL2RG	MUD	PBSC	CD34+	**RIC** (FLU148/TREO36)	ALEM	MMF	-	-
15	13	IL2RG	Brother	BM	-	-	-	CSA	-	-
16	8	RAG1	Mother	BM	-	-	-	-	I° (skin)	+
17	7	ZAP70	Father	PBSC	E-rosetting	-	-	CSA	-	-
26	3,5	ADA	Brother	BM	-	-	-	PDN	I° (skin)	-
30	5,5	Artemis	MUD	PBSC	CD34+	**RIC** (FLU150/TREO36)	ALEM	MMF	II° (skin)	-
33	6	Unknown	Sister	BM	-	-	ALEM	CSA, PDN	I° (skin)	-

MA: myeloablative, RTC: reduced-toxicity conditioning, CY: cyclophosphamide, TT: thiotepa, FLU: fludarabin, BU: busulfan, TREO: treosulfan, ALEM: alemtuzumab, ATG: anti-thymocyte globulin; MUD matched unrelated donor; CSA: cyclosporin A, MMF: mycofenolate mofetil; PDN: prednisolone; IS: immunosuppressive; *Multiple HSCT with and without conditioning; [%] Re-HSCT when 20 y old (CD34+, ATG); †Patient HLA compatible with mother. Conditioning: bracketed abbreviations and numbers indicate cumulative drug dosis.
